# A pH-Responsive Ti-Based Local Drug Delivery System for Osteosarcoma Therapy

**DOI:** 10.3390/jfb15100312

**Published:** 2024-10-21

**Authors:** Qinle Xiao, Changjun Wan, Zhe Zhang, Hui Liu, Pingting Liu, Qianli Huang, Dapeng Zhao

**Affiliations:** 1 College of Biology, Hunan University, Changsha 410082, China; 2 State Key Laboratory of Powder Metallurgy, Central South University, Changsha 410083, China

**Keywords:** titanium implant, local drug delivery, pH-responsive drug release, osteosarcoma

## Abstract

Osteosarcoma is one of the major bone cancers, especially for youngsters. The current treatment usually requires systemic chemotherapy and the removal of bone tumors. Titanium (Ti)-based implants can be modified as local drug delivery (LDD) systems for controllable and localized chemotherapeutic drug release. In this work, a pH-responsive Ti-based LDD prototype was designed by introducing polydopamine (PDA) to release doxorubicin (DOX) around osteosarcoma cells with low pH. Fourier transform infrared spectroscopy (FTIR), scanning electron microscopy (SEM), and a contact angle meter were applied for surface characterization. Both direct and indirect cell culture modes were performed for biocompatibility and biofunction assessments. The results indicate that the Ti-based LDD prototype exhibits significant pH-dependent DOX release. The cumulative release can reach up to approximately 40% at pH = 6.0 after 72 h, but only around 20% at pH = 7.4. The Ti-based LDD implant shows good biocompatibility with approximately 93% viability of MC3T3 cells after direct culture in vitro for 24 h. Both direct and indirect culture modes verify the good anti-osteosarcoma function of the LDD implant, which should be attributed to the pH-responsive release of DOX.

## 1. Introduction

Osteosarcoma is the most common primary malignant bone cancer with an incidence of 4 per million people aged 0–24 years or over 60, and 1.7 per million people aged between 25 and 59 years old [1]. The current treatment of osteosarcoma combines chemotherapy and surgical resection of disease, i.e., the standard preoperative chemotherapy, the surgical resection followed by implanting of replacement materials, and the long-term postoperative chemotherapy [2]. However, the current treatment still needs to be improved. Firstly, the systemic delivery of chemotherapeutic drugs, including doxorubicin (DOX), cisplatin, and methotrexate, often leads to severe side effects on healthy tissue and vital organs, and the short half-lives of many chemotherapeutic drugs inevitably result in frequent administration and high total doses [3,4]. Secondly, residual tumor cells usually survive around bone tissues after surgery, which can lead to a relapse of osteosarcoma [5]. Thirdly, surgical intervention often results in large bone defects, which can hardly be healed by patients themselves [6].

Local drug delivery (LDD) implant systems have emerged as a potential alternative to traditional systemic administration of drugs for the clinical treatment of various diseases. LDD systems can provide controlled, localized, and sustained drug release at the site of interest. This capability significantly enhances therapeutic efficacy while minimizing side effects [7]. Titanium (Ti) and its alloys are widely used as bone replacement materials due to their good comprehensive mechanical properties, excellent corrosion resistance, and great biocompatibility [8,9]. Most Ti-based implants currently serve as mechanical supports in clinical practice [10]. However, increasing clinical requirements for biofunctions, e.g., osseointegration, anti-bacteria, and anti-cancer, promote the development of new functionalized Ti-based implants. Recently, many Ti-based LDD systems have been developed [11,12,13]. Among them, titania nanoarrays (TNAs) fabricated by anodization on the surface of Ti-based implants are considered excellent drug carriers [14,15] due to their high biocompatibility [16], enormous specific surface area [17], and good stability [18]. For instance, Gagandeep et al. [19] developed an LDD system by loading TNF-related apoptosis-inducing ligand (TRAIL) onto TNAs. The TRAIL-TNA system showed significant inhibition of breast cancer proliferation. Wei et al. [20] incorporated BMP-2/macrophage exosomes onto TNAs, and the system activated autophagy during osteogenic differentiation. However, in these studies, drugs were loaded directly onto TNAs, and thus the drug release could not be tailored, so challenges related to drug delivery and sustained release remain to be addressed for effective clinical applications.

Polydopamine (PDA) has attracted extensive attention for biomedical applications because of its good biocompatibility [21], strong adhesive properties to various kinds of materials [22], and sensitive pH-responsiveness [23]. Some previous studies verified the efficiency of PDA-modified TNAs for improved drug delivery. The anticoagulant drug bivalirudin (BVLD) was loaded onto PDA-coated TNAs, and the BVLD-PDA@TNAs system showed significantly enhanced hemocompatibility compared with flat Ti and bare TNAs [24]. PDA was also used to modify vancomycin-loaded TNAs in order to achieve better and more controllable antibacterial ability [25].

DOX is still one of the leading drugs for osteosarcoma chemotherapy due to its excellent anticancer activity and low cost [26]. Therefore, it was used as a model drug in this work, and PDA was employed onto TNAs for the pH-responsive release of DOX. This research aims to provide an in-depth explanation of the drug release mechanism of a PDA-coated TNA LDD system and to make a comprehensive in vitro evaluation of the biocompatibility and the pH-responsive anti-cancer biofunction of the LDD prototype by using both direct and indirect culture modes.

## 2. Materials and Methods

### 2.1. Raw Materials

The commercial pure titanium slices (CP-Ti) (99.6% purity, Guanyue Metal Co., Ltd., Guangzhou, China) were prepared specifically for experiments. The dimension of the slices was 10 mm × 10 mm × 2.5 mm. The surfaces of the titanium slices were meticulously polished using silicon carbide sandpaper with grit sizes of 360, 400, 800, 1000, and 2000 in sequence. Subsequently, the slices underwent a thorough cleaning process involving acetone (Sinopharm Chemical Reagent Co., Ltd., Shanghai, China), absolute ethanol (Sinopharm Chemical Reagent Co., Ltd.), and distilled water in an ultrasonic cleaner for 15 min each. Following the cleaning procedure, the specimens were dried using an electric blast drying oven.

### 2.2. TNA Preparation

TNAs were fabricated by anodization, utilizing graphite foil as the counter cathode in a 300 mL electrolyte solution comprising 97 vol % ethylene glycol (Sinopharm Chemical Reagent Co., Ltd.), 3 vol % deionized water, and 0.5 wt % NH_4_F (Sinopharm Chemical Reagent Co., Ltd.). The anodization process involved magnetic stirring at a constant speed for 50 min with an anodization voltage of 50 V [27]. Upon completion of the anodization, the specimens were promptly rinsed with deionized water, subjected to ultrasonication in ethylene glycol for 10 min, and then washed with distilled water. The TNAs were subsequently dried in the electric blast drying oven and stored in glass desiccators, and the samples obtained were named TNA.

The obtained TNA was immersed in 0.5 M NaOH solution (Sinopharm Chemical Reagent Co., Ltd.) for 30 min for hydroxylation. After soaking, the samples were washed three times and then dried. The dried sample was immersed in a solution containing 2 mg/mL dopamine hydrochloride (DA, Sigma-Aldrich, St. Louis, MA, USA) configured with 10 mM, pH 8.5 Tris-HCl buffer in a dark environment at room temperature. At the end of the reaction, the samples were washed three times with plenty of deionized water and then dried, and the samples obtained were named TNA-PDA.

### 2.3. Drug Loading

The DOX (Sangon Biotech Co., Ltd., Shanghai, China) masterbatch was first prepared at a concentration of 1 mg/mL. The DOX masterbatch was diluted with deionized water to obtain a DOX solution of 0.1 mg/mL. Then, 50 μL of this concentration was applied to the surface of the TNA and TNA-PDA samples using a pipette gun and gently tapped to ensure that the liquid beads covered the sample surface evenly. The samples were then gently transferred to an oven for drying. After the drug was completely dry, 50 μL of deionized water was pipetted onto the sample surface using a pipette gun, spread evenly, and transferred to an oven for drying. After four sequential loading and drying steps, the TNA and TNA-PDA samples were loaded with 20 μg of drug, and the resulting samples were named TNA-DOX and TNA-PDA-DOX [28,29].

### 2.4. Surface Morphology and Properties

Surface functional groups of the samples were detected by Fourier transform infrared spectroscopy (FTIR, NEXUS670, Nicolet, Madison, WI, USA) with a scan range of 2500–500 cm^−1^. The surface morphology of the different samples was observed by field emission scanning electron microscopy (FE-SEM, Helios Nanolab G3 UC, FEI Co., Hillsboro, OR, USA) in the secondary electron mode. Surface wettability was determined using a video-enabled contact angle meter (SDC-200 S, ShengDing, Jiaxing, China) by testing the static water contact angle of different samples using the static drop method, with three randomly selected locations for each measurement.

### 2.5. Drug Release

To investigate the DOX release behavior of TNA-DOX and TNA-PDA-DOX in different pH environments, samples were immersed in 15 mL centrifuge tubes containing 4 mL PBS (pH = 7.4, 6.5, or 6.0), and the pH was adjusted using 1 M hydrochloric acid and 1 M sodium hydroxide solutions, with three parallel groups in each group. All groups were then incubated at 37 °C in a light-protected thermostat. Measurements were taken hourly during the first 6 h period, defined as the initial phase of drug release. Subsequent time points of 24, 48, and 72 h were used to assess the slow phase of drug release. Drug release at each time point was measured by fluorescence spectrometry and indirect methods using a fluorescence spectrometer with the excitation light wavelength set at 510 nm and the absorbance value read at 558 nm.

To determine the drug concentration corresponding to the absorbance measured at each time point, a drug standard curve corresponding to the absorbance of a known drug concentration must be generated. DOX solutions with concentrations of 0.1, 0.2, 0.3, 0.4, 0.5, 0.75, and 1 μg/mL were obtained by dilution and the excitation light of 510 nm was set to read the absorbance values of different concentrations of PBS solutions at 558 nm. The data were analyzed using linear regression fitting to obtain the standard curve equation for the drug. The drug standard curve can be used to determine the concentration of drug in PBS obtained from the collection at each time point.

In addition, to determine the decomposition of PDA in a slightly acidic environment, the release of DA from TNA-PDA and TNA-PDA-DOX was also characterized. Samples were immersed in 4 mL PBS (pH = 6.0). The absorbance was measured at 280 nm by using UV-Vis spectroscopy. Time points of 24, 48, and 72 h were used, and at least three samples were tested for each configuration.

### 2.6. Cell Culture

MG-63 (human osteosarcoma cells) and MC3T3 (mouse osteoblast cell) cell lines were purchased from ATCC in the USA, and cell culture was performed under strict aseptic conditions. All cells were cultured in Dulbecco’s modified Eagle’s medium (DMEM, Gibco, Waltham, MA, USA) with high glucose and pyruvate content supplemented with 10% (*v*/*v*) fetal bovine serum (Gibco, USA) and 1% (*w*/*v*) penicillin/streptomycin (Gibco, USA) at 37 °C in a 5% CO_2_ incubator.

### 2.7. Cell Viability Assay

**Figure 1:**
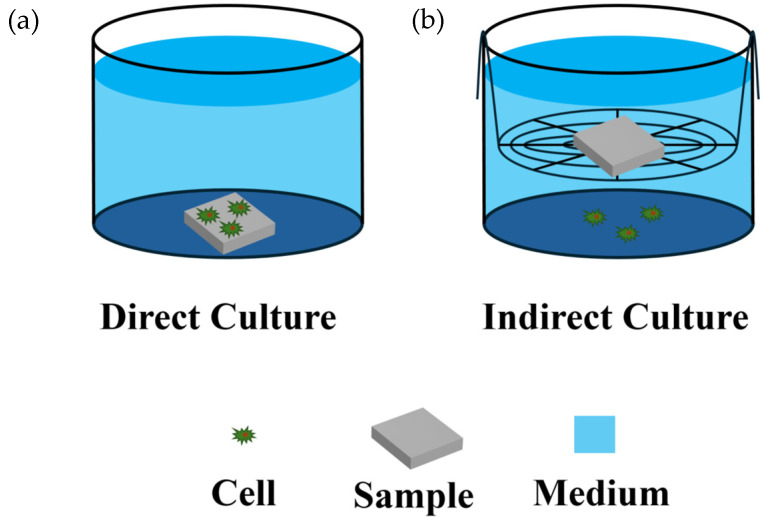
Cell culture modes: (**a**) Direct culture, (**b**) Indirect culture.

The cell viability of MC3T3 and MG-63 cells was assessed by either direct or indirect culture modes as presented in Figure 1.

The direct cell-sample co-culture was performed to determine the interaction between cells and sample surfaces, and the MTT (Sangon Biotech (Shanghai) Co., Ltd.) assay was used for the viability assay of MC3T3 and MG-63 cells. Briefly, 100 μL of cell suspension (2 × 10^4^ cells/well) was inoculated onto the samples (Figure 1a), and the plate was incubated for 1 h to ensure that the cells were firmly attached to the surface of the samples. Then, the total medium of the 24-well plate was fixed to 2 mL. After a 24-h incubation period, 500 μL of MTT and cell culture medium mixture, prepared at a 1:10 volume ratio (*v*/*v*), was added to each well. The plates were then returned to the incubator for an additional 4 h of incubation. The medium was then carefully removed and 500 μL of DMSO (Sinopharm Chemical Reagent Co., Ltd.) was added, placed on a shaker for 30 min, and then pipetted into a 96-well plate, and the absorbance value (OD) of each well was measured at 490 nm using a microtiter plate.

The viability of MG-63 cells in indirect cell-sample co-culture was examined using the CCK-8 (Biosharp Life Sciences Co., Ltd., Hefei, China) assay, in which MG-63 cells were first inoculated onto cell slips (JET CellSlip^TM^, Guangzhou JET Bio-Filtration Co., Ltd., Guangzhou, China) with an inoculum of 2 × 10^4^ per cell slip. After the cells had grown for 12 h, the medium was changed to a medium with a different pH (the pH of the medium was previously adjusted with 1 M HCl and 1 M NaOH and filtered with a 0.22 μm filter tip to remove bacteria), then the samples were placed on the wire racks with the bottom not touching the cells, as shown in Figure 1b, and three parallel groups with different pH values were established. After incubation for 24 h, the wire rack and samples were removed, and 500 μL of a mixture of CCK-8 and cell culture medium (1:10, *v*/*v*) was added to each well, which was incubated at 37 °C for 1 h. The plate was gently shaken and then pipetted into a 96-well plate, and the absorbance (OD) of each well was measured at 450 nm using a microtiter plate.

### 2.8. Cell Morphology

Cells were directly incubated with different samples for one day, fixed with 3% glutaraldehyde (Sinopharm Chemical Reagent Co., Ltd.), and dehydrated through an ethanol gradient. The samples were dried and sprayed with gold before visualization by FE-SEM.

### 2.9. Live/Dead Staining Assay

Cells were stained live/dead after one day of indirect or direct incubation with different samples. The staining procedure was performed with reference to the assay kit. The cells were observed using a laser confocal microscope (Olympus FV1200, Tokyo, Japan) after staining was completed. Calcein-AM (green fluorescence) and PI (red fluorescence) (Beyotime Biotech Inc., Haimen, China) were used to specifically stain live and dead tumor cells, respectively. Live and dead cells were counted and analyzed using ImageJ software (Version 1.54d, 2023).

### 2.10. Cellular Uptake Studies on Drug

In the indirect contact culture model, cells were incubated with TNA-PDA-DOX samples for 6 h. After incubation, the medium was discarded, and the cells were washed with PBS (Biosharp Life Sciences Co., Ltd.). 4% paraformaldehyde (Sinopharm Chemical Reagent Co., Ltd.) was added for fixation, followed by Triton X-100 for membrane disruption and DAPI (Beijing Solarbio Science & Technology Co., Ltd., Beijing, China) for staining. The cells were then observed by laser confocal microscopy, with DAPI and DOX excited at 405 and 485 nm, respectively. The fluorescence intensity and co-localization of DOX in the cells were analyzed using ImageJ software. For co-localization, three randomly selected regions from each image were analyzed, and scatter plots were generated. The corresponding Pearson coefficients were calculated as a reference for co-localization. The Pearson coefficient reflects the overlap of the two fluorescent dyes and ranges from 1 to −1, where 1 represents a perfect positive correlation, −1 represents a negative correlation, and zero represents no correlation [30]. The slope of the scatter plot reflects the fluorescence ratio of the two channels [31].

### 2.11. Statistical Analyses

In this work, at least three samples were used for each experimental group and all quantitative results were presented as mean and standard deviation. The one-way analysis of variance (ANOVA) was performed using SPSS software (Version 27, 2009) to indicate statistically significant differences between groups at *p* values less than or equal to 0.05 (*: *p* ≤ 0.05).

## 3. Results

### 3.1. Characterization of Samples

**Figure 2:**
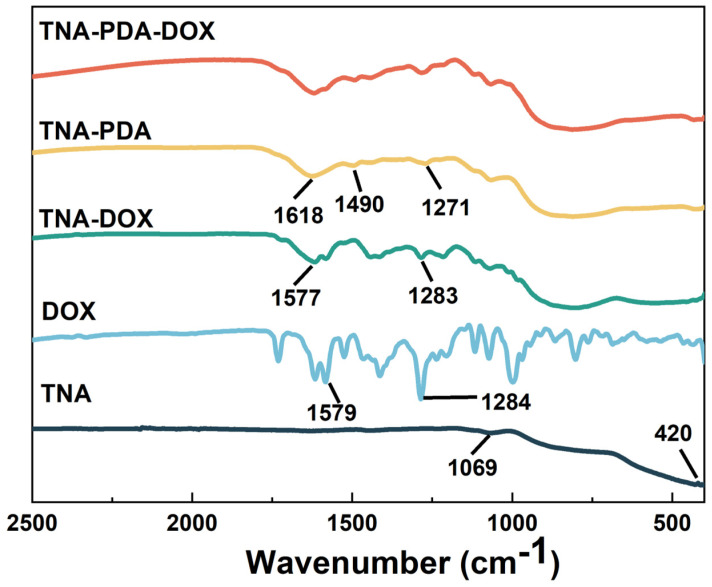
Infrared spectra of TNA, DOX, TNA-DOX, TNA-PDA, and TNA-PDA-DOX.

The FTIR spectra of samples are shown in Figure 2. The characteristic peaks of TNA appear at 1069 cm^−1^ and 420 cm^−1^, which are related to the bending vibrations of H-O-Ti [32] and the lattice vibrations of TiO_2_ [33], respectively, and these characteristic peaks are also found in the infrared spectra of TNA-DOX, TNA-PDA, and TNA-PDA-DOX. As for TNA-PDA, the characteristic peak at 1271 cm^−1^ is attributed to the stretching vibration of the phenolic C–OH groups in polydopamine [34], and the characteristic broad PDA signals are detected in the region of 1618–1490 cm^−1^, which are assigned to the stretching vibration of the aromatic ring, the bending vibration of N-H, the N-H shearing vibration of the amide group, and the C-O vibration [35]. The characteristic peaks at 1283 cm^−1^ (TNA-DOX) and 1284 cm^−1^ (DOX) are related to the stretching of alcoholic O–H groups [36], and those at 1577 cm^−1^ (TNA-DOX) and 1579 cm^−1^ (DOX) can be attributed to the aromatic nature (benzene ring) [37]. In addition, the characteristic peaks of PDA and DOX can also be found in the spectrum of TNA-PDA-DOX.

**Figure 3:**
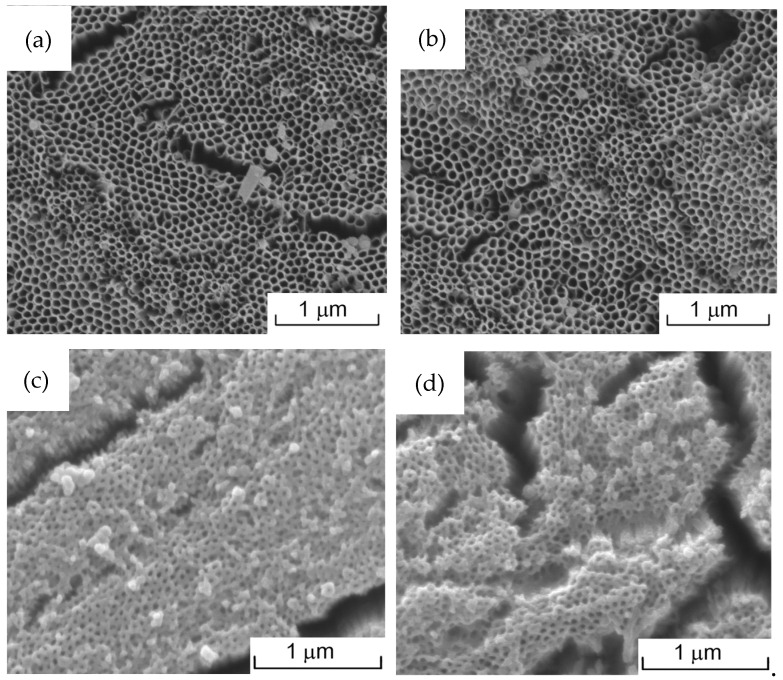
SEM images of (**a**) TNA, (**b**) TNA-DOX, (**c**) TNA-PDA, and (**d**) TNA-PDA-DOX.

Figure 3 shows the surface morphologies of TNA, TNA-DOX, TNA-PDA, and TNA-PDA-DOX. TNA presents a well-aligned nanotubular structure with an inner diameter of 53 ± 5 nm after anodization for 50 min at 30 V, as shown in Figure 3a. Compared with TNA, TNA-PDA exhibits similar tightly arranged nanotube arrays but reduced inner diameter (43 ± 6 nm). The loading of DOX does not change the surface morphology, since Figure 3b,d present almost the same nanotubular structures as Figure 3a and Figure 3c, respectively.

**Figure 4:**
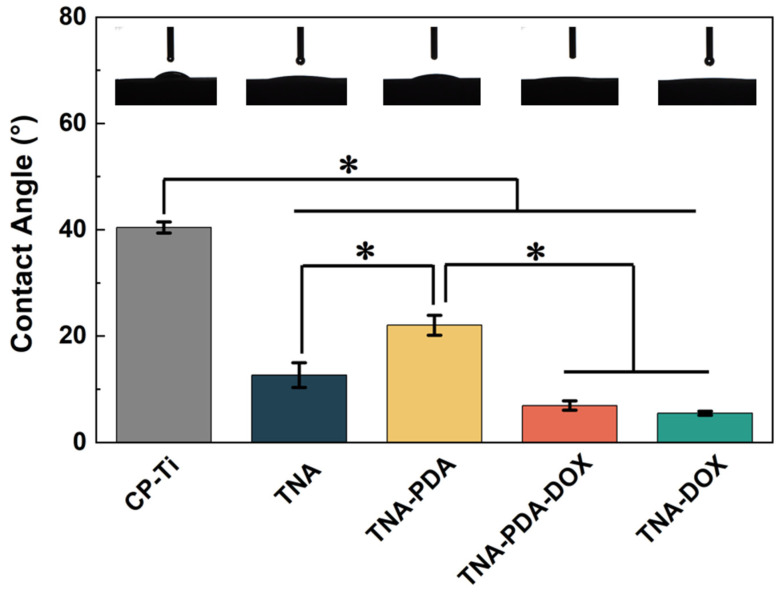
Water contact angles of CP-Ti, TNA, TNA-DOX, TNA-PDA, and TNA-PDA-DOX. *: *p* < 0.05.

The wettability of different sample surfaces was evaluated by measuring static water contact angles, as shown in Figure 4. Compared with other anodized groups, the CP-Ti group shows the highest contact angle (40 ± 1°). Similar to previous studies [16,17,38], the samples after anodization (i.e., TNA, TNA-DOX, TNA-PDA, and TNA-PDA-DOX) show good hydrophilicity with static water contact angles lower than 25°. The modification of PDA increases contact angles from 13 ± 2° (TNA) to 22 ± 2° (TNA-PDA). In contrast, the introduction of DOX leads to super-hydrophilicity on TNA-DOX and TNA-PDA-DOX.

### 3.2. In Vitro Drug Release of DOX

**Figure 5:**
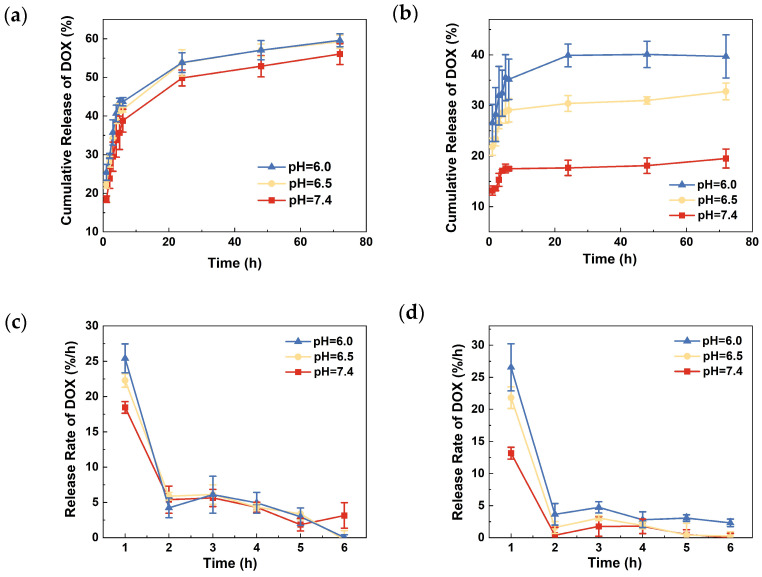
The drug release profiles of (**a**) TNA-DOX and (**b**) TNA-PDA-DOX at pH = 7.4, 6.5, and 6.0, and the release rates of DOX from (**c**) TNA-DOX and (**d**) TNA-PDA-DOX at pH = 7.4, 6.5, and 6.0 within the first 6 h.

Figure 5 presents the drug release profiles of TNA-DOX and TNA-PDA-DOX in PBS with different pH values (pH = 7.4, 6.5, and 6.0). As shown in Figure 5a, the DOX release from TNA-DOX at different pH values shows similar trends, i.e., a burst release during the initial 6 h followed by a decreased release of DOX from 24 h to 72 h. About 55–60% of loaded DOX is released regardless of pH level after 72 h. In contrast, TNA-PDA-DOX shows an obviously pH-responsive drug release behavior as presented in Figure 5b. The cumulative DOX release from TNA-PDA-DOX is always higher at a lower pH level. After releasing for 72 h, the cumulative release amount of DOX can reach 19.5 ± 1.9%, 32.8 ± 1.6%, and 39.7 ± 4.3% at pH = 7.4, 6.5, and 6.0, respectively. To present more details of the burst release, the cumulative release rates of TNA-DOX and TNA-PDA-DOX within the initial 6 h are shown in Figure 5c,d, respectively. The rates of DOX release from TNA-DOX at the three pH values do not show significant differences from the 2nd hour. However, the release rates from TNA-PDA-DOX at pH = 6.0 are higher than those at pH = 6.5 and 7.4 (see Figure 5d). It is important to note that TNA-PDA-DOX shows about 50–90% and 40–80% lower release rates than TNA-DOX at pH = 7.4 and 6.5, respectively, but at pH = 6.0, the drug release from the former samples is only 10–40% slower than that from the latter specimens.

In addition, to verify the stability of PDA coatings on TNA-PDA and TNA-PDA-DOX in a slightly acidic environment, the DA release was also tested at pH = 6.0 for 24, 48, and 72 h, and the release profiles are shown in Figure S1. It is obvious that the decomposition of PDA is very low for both TNA-PDA (0.01–0.03%) and TNA-PDA-DOX (0.02–0.04%).

The first-order model (see Equation (1)) [39], Higuchi model (see Equation (2)) [40], zero-order model (see Equation (3)) [41], Korsmeyer-Peppas model (see Equation (4)) [42], and Gallagher-Corrigan release model [43] (see Equation (5)) were employed to better understand the release kinetics of the samples:

(1) $$f_{t}=1-e^{-K_{i}t}$$

(2) $$f_{t}=K_{h}t^{1/2}$$

(3) $$f_{t}=K_{j}t$$

(4) $$f_{t}=K_{l\;}t^{n}$$

(5) $$f_{t}=f_{max}\left(1-e^{-k_{1}t}\right)+\left(f_{max}-f_{b}\right)\left(\frac{e^{k_{2}t-k_{2}t_{max}}}{1+e^{k_{2}t-k_{2}t_{max}}}\right)$$

where *f_t_* is the accumulative fraction of released drug at time *t*, *K_i_* is the first-order kinetic constant, *K_h_* is the Higuchi dissolution constant, *K_j_* is the zero-order kinetic constant, *K_l_* is the Korsmeyer-Peppas constant, *n* is the release exponent, *f_b_* is the fraction of released drug during the first burst stage, *k_1_* and *k_2_* are the kinetic constants for the first and second stages of release, respectively, and *t_max_* is the time to maximum drug release rate. In this work, *t_max_* should be 1 for all samples as shown in Figure 5c,d.

**Figure 6:**
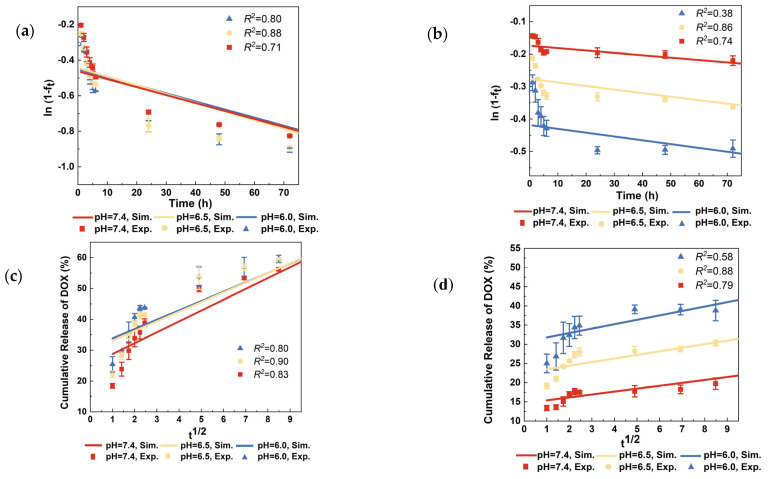

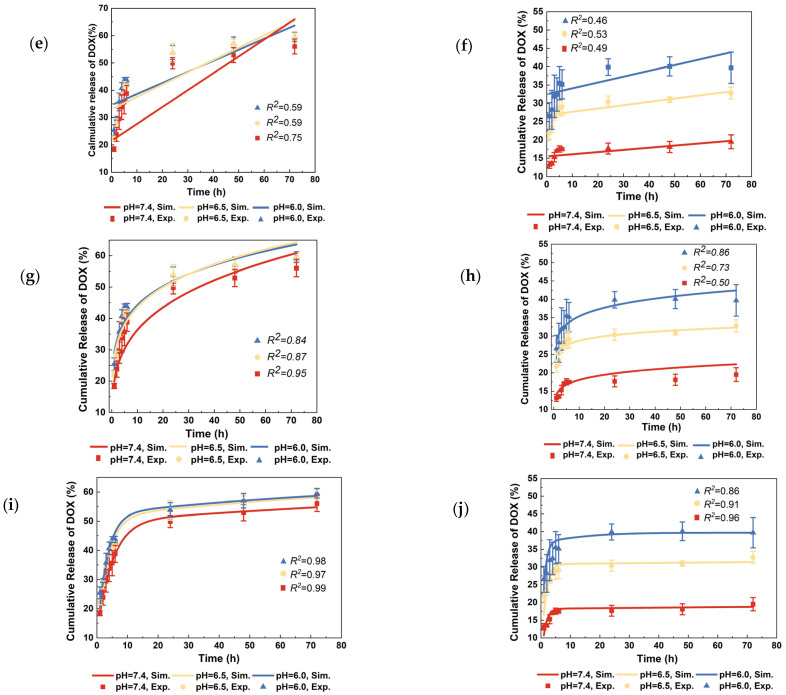
The experimental data and the corresponding simulated curves from (**a**) TNA-DOX (by using the first-order model); (**b**) TNA-PDA-DOX (by using the first-order model); (**c**) TNA-DOX (by using Higuchi model); (**d**) TNA-PDA-DOX (by using Higuchi model); (**e**) TNA-DOX (by using the zero-order model); (**f**) TNA-PDA-DOX (by using the zero-order model); (**g**) TNA-DOX (by using Korsmeyer-Peppas model); (**h**) TNA-PDA-DOX (by using Korsmeyer-Peppas model); (**i**) TNA-DOX (by using Gallagher-Corrigan release model); (**j**) TNA-PDA-DOX (by using Gallagher-Corrigan release model) at pH = 7.4, 6.5, or 6.0.

Figure 6 shows the experimental data and the corresponding simulation curves from TNA-DOX and TNA-PDA-DOX by using the five above-mentioned models. After fitting, it is found that the first-order model, Higuchi model, zero-order model, and Korsmeyer-Peppas model show relatively poor fitting. In contrast, the Gallagher-Corrigan model with the description of two-stage drug release exhibits much better simulation results. The first stage corresponds to the burst release of the drug, and the second stage corresponds to the slow release. Table 1 summarizes the release fitting parameters *k_1_* and *k_2_* for TNA-DOX and TNA-PDA-DOX under different pH conditions. According to Table 1, TNA-PDA-DOX shows lower *k_1_* and *k_2_* than TNA-DOX at the same pH value.

### 3.3. Biocompatibility

**Figure 7:**
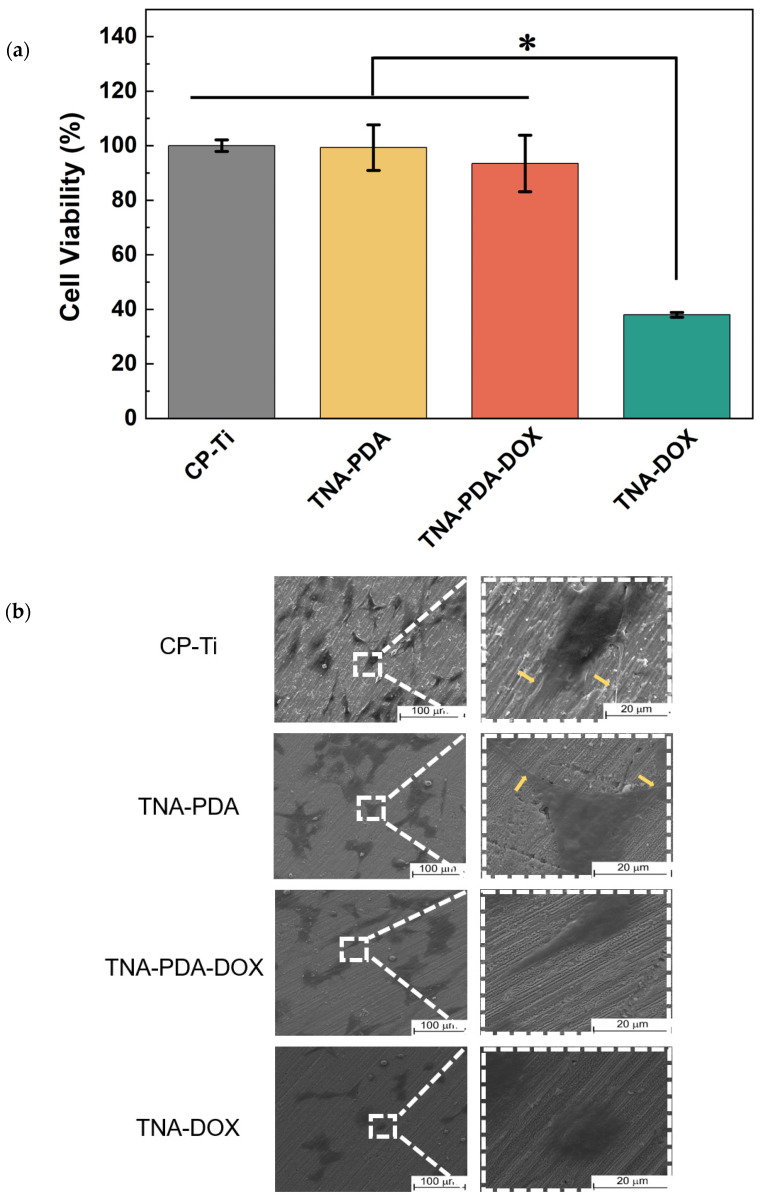

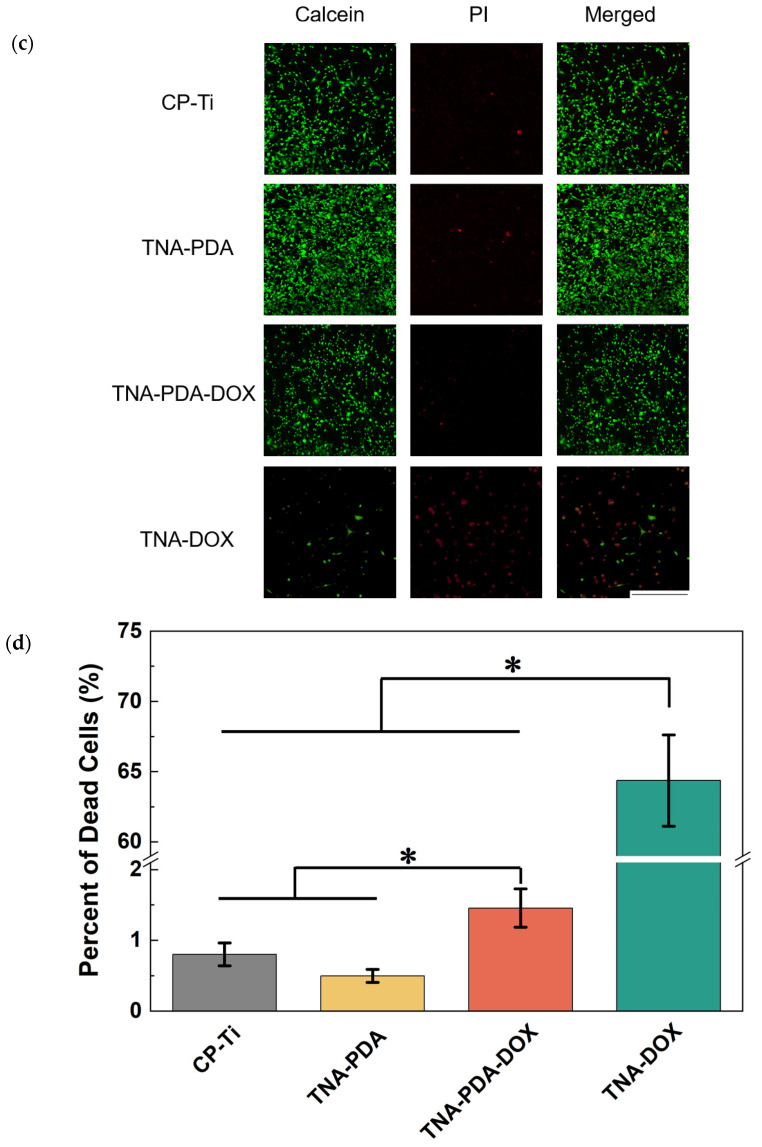
The cell viability and morphology of MC3T3 cultured for 24 h on CP-Ti, TNA-PDA, TNA-PDA-DOX, and TNA-DOX samples. (**a**) MTT assay results at 490 nm; (**b**) FE-SEM images (the yellow arrows mark the filopodia of MC3T3 cells); (**c**) live/dead fluorescence staining images (scale bar: 200 μm); (**d**) the percents of dead cells calculated based on live/dead staining results. *: *p* < 0.05.

Figure 7 shows the cell viability and morphology of MC3T3 seeded on CP-Ti, TNA-PDA, TNA-PDA-DOX, and TNA-DOX samples for 24 h. Figure 7a presents the cell viability cultured directly on the four configurations by MTT assay. The cell viabilities on TNA-DOX (~38%) samples are significantly lower than those on CP-Ti, which acts as the control group, TNA-PDA (~99%), and TNA-PDA-DOX (~93%). SEM images of MC3T3 cells on the four groups of samples are presented in Figure 7b. After being cultured for 24 h, most cells on CP-Ti exhibit typical spindle shapes with some filopodia marked with yellow arrows. Both elongated-spindle-shaped and flat-star-shaped MC3T3 cells are presented on TNA-PDA and TNA-PDA-DOX samples, whereas there are many flat cells without obvious filopodia on TNA-DOX samples. The live/dead staining images, as presented in Figure 7c, show that the numbers of live MC3T3 cells cultured on CP-Ti, TNA-PDA, and TNA-PDA-DOX specimens are significantly higher than that on TNA-DOX, and many more dead cells are observed on the latter samples than the former three groups. In order to quantify the cell viability of MC3T3 on the samples, the percentages of dead cells on the four groups were calculated according to live/dead staining results as shown in Figure 7d. The dead cell percentages on CP-Ti, TNA-PDA, and TNA-PDA-DOX groups are all below 2%; nonetheless, that on TNA-DOX is about 65%.

### 3.4. Anti-Cancer Evaluation In Vitro

**Figure 8:**
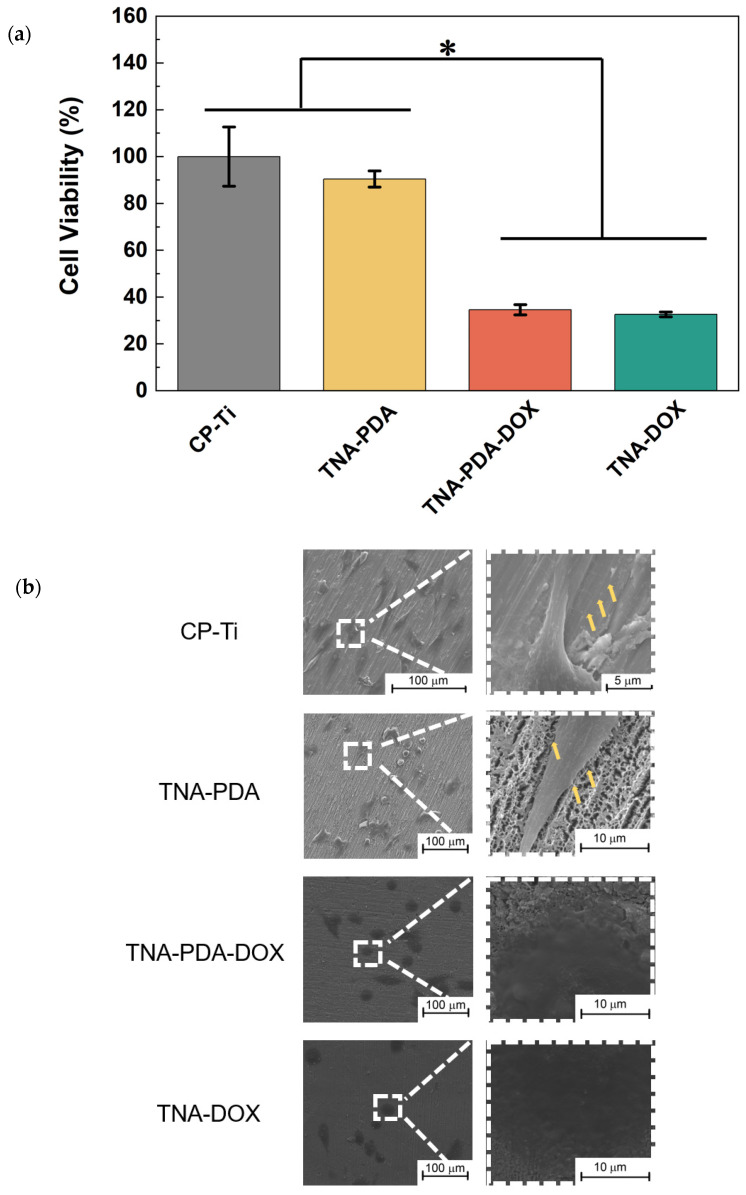

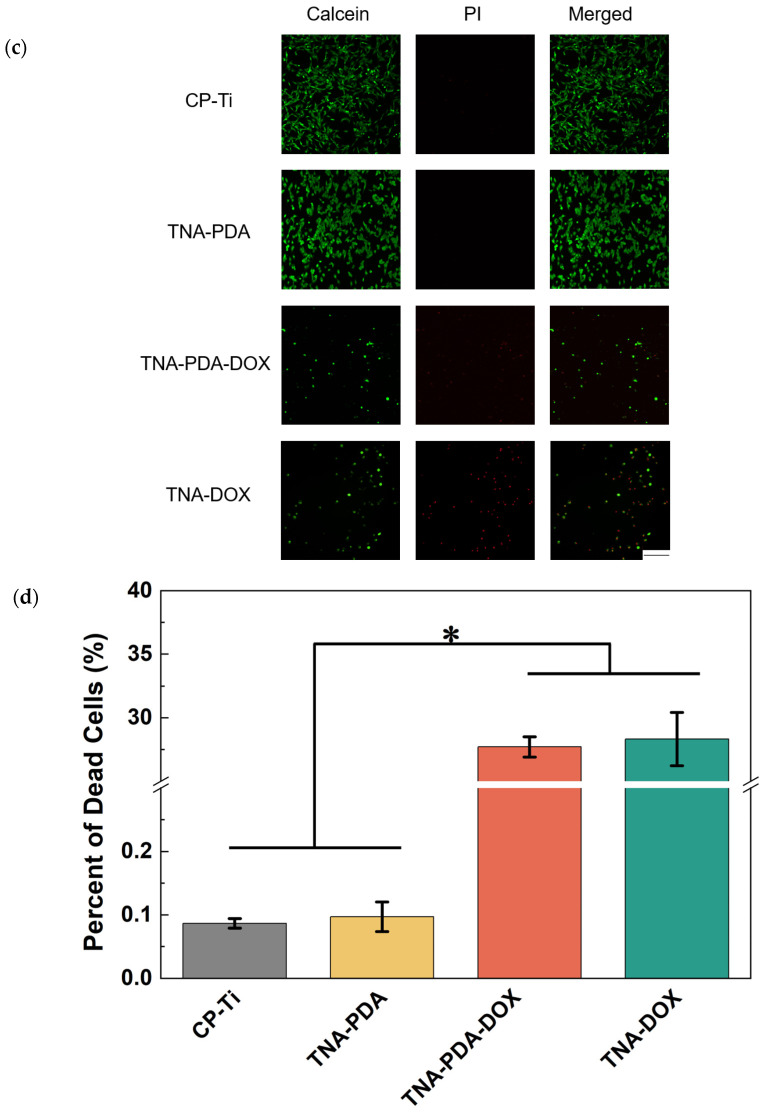
The cell viability and morphology of MG63 cultured for 24 h on CP-Ti, TNA-PDA, TNA-PDA-DOX, and TNA-DOX samples. (**a**) MTT assay results at 490 nm; (**b**) FE-SEM images (the yellow arrows mark the filopodia of MG63 cells); (**c**) live/dead fluorescence staining images (scale bar: 200 μm); (**d**) the percentages of dead cells calculated based on live/dead staining results. *: *p* < 0.05.

Figure 8 shows the anti-cancer behavior of CP-Ti, TNA-PDA, TNA-PDA-DOX, and TNA-DOX, on which MG63 cells were directly cultured for 24 h. As presented in Figure 8a, the MTT results show that the cell viability on TNA-PDA is not significantly different from that on CP-Ti. The absorbances of TNA-PDA-DOX and TNA-DOX groups are only 40% of that of the CP-Ti group. The SEM images as presented in Figure 8b show different cell morphologies of MG63 on the four groups of samples. Generally, the cells on CP-Ti and TNA-PDA samples are mostly spindle-shaped. Many filopodia (marked with yellow arrows) and ruffled cell surfaces are observed. In contrast, the cells on TNA-PDA-DOX and TNA-DOX are usually round and flat without obvious filopodia. Figure 8c,d perform the qualitative analyses of the efficacy of the samples in direct contact with MG63 cells and the quantitative proportions of dead cells on the four configurations by using live/dead staining, respectively. CP-Ti and TNA-PDA show comparable high cell density with very few dead cells, but on TNA-PDA-DOX and TNA-DOX samples, there are many fewer live cells, and significantly higher percentages of dead cells than those on the other two configurations.

**Figure 9:**
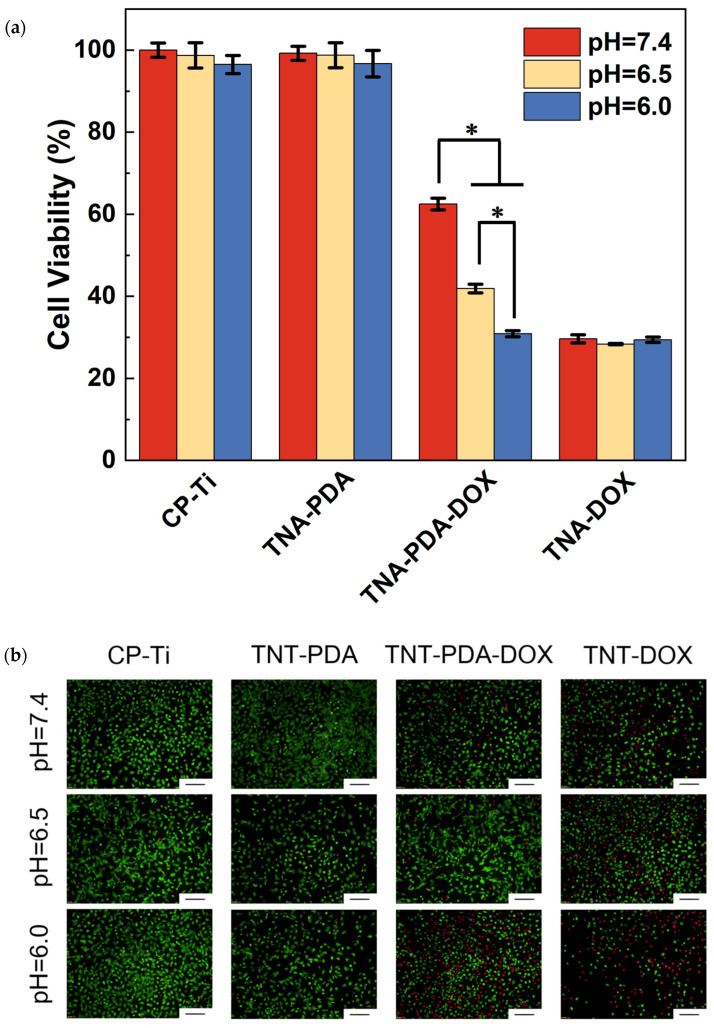

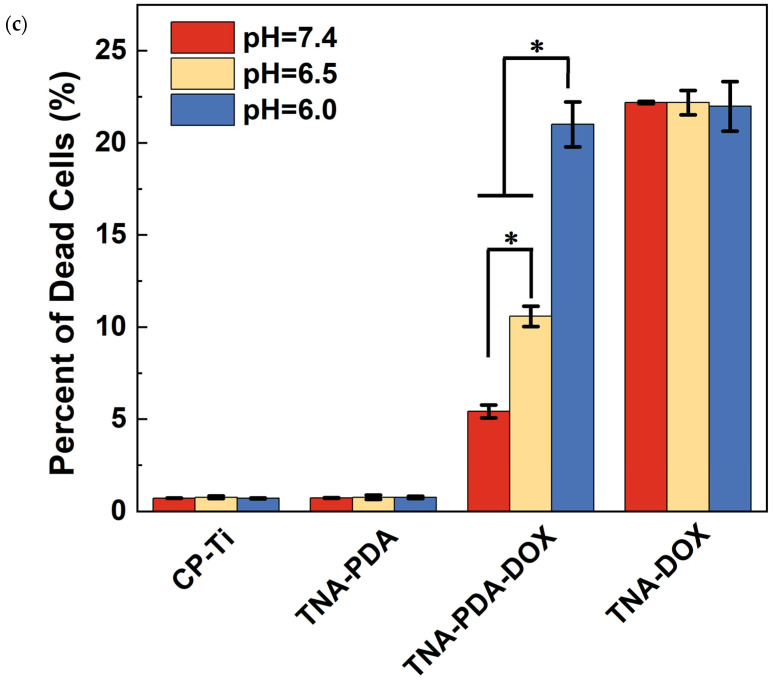
The cell viability of MG63 indirectly cultured for 24 h with CP-Ti, TNA-PDA, TNA-PDA-DOX, and TNA-DOX samples at pH = 7.4, 6.5, or 6.0. (**a**) CCK-8 assay results at 450 nm; (**b**) live/dead fluorescence staining images (scale bar: 200 μm); (**c**) the percentages of dead cells calculated based on live/dead staining results. *: *p* < 0.05.

The influence of pH values of the culture medium on the anti-cancer behavior of samples was evaluated by the indirect culture with MG63 cells via CCK-8 and live/dead staining, as presented in Figure 9. Figure 9a shows that at the three pH values (pH = 7.4, 6.5, and 6.0), the cell viabilities in CP-Ti, TNA-PDA, and TNA-DOX groups are 96–100%, 96–99%, and 28–30%, respectively. However, the cell viability in the TNA-PDA-DOX group is highly pH-dependent, i.e., about 62% at pH = 7.4, about 42% at pH = 6.5, and about 31% at pH = 6.0. Similarly, the live/dead staining images (see Figure 9b) present that a great number of live spindle-shaped MG63 cells were observed at all pH values in the CP-Ti and TNA-PDA groups. There are many red spots, showing dead cells, in the TNA-DOX group at all three pH values. It is important to note that with decreasing pH, not only does the number of dead cells increase, but the cell morphology of MG63 also tends to be round instead of elongated spindle-shaped in the TNA-PDA-DOX group. Furthermore, the quantified percentage of dead cells as shown in Figure 9c also clearly exhibits the pH-dependent anti-osteosarcoma behavior of the TNA-PDA-DOX group.

**Figure 10:**
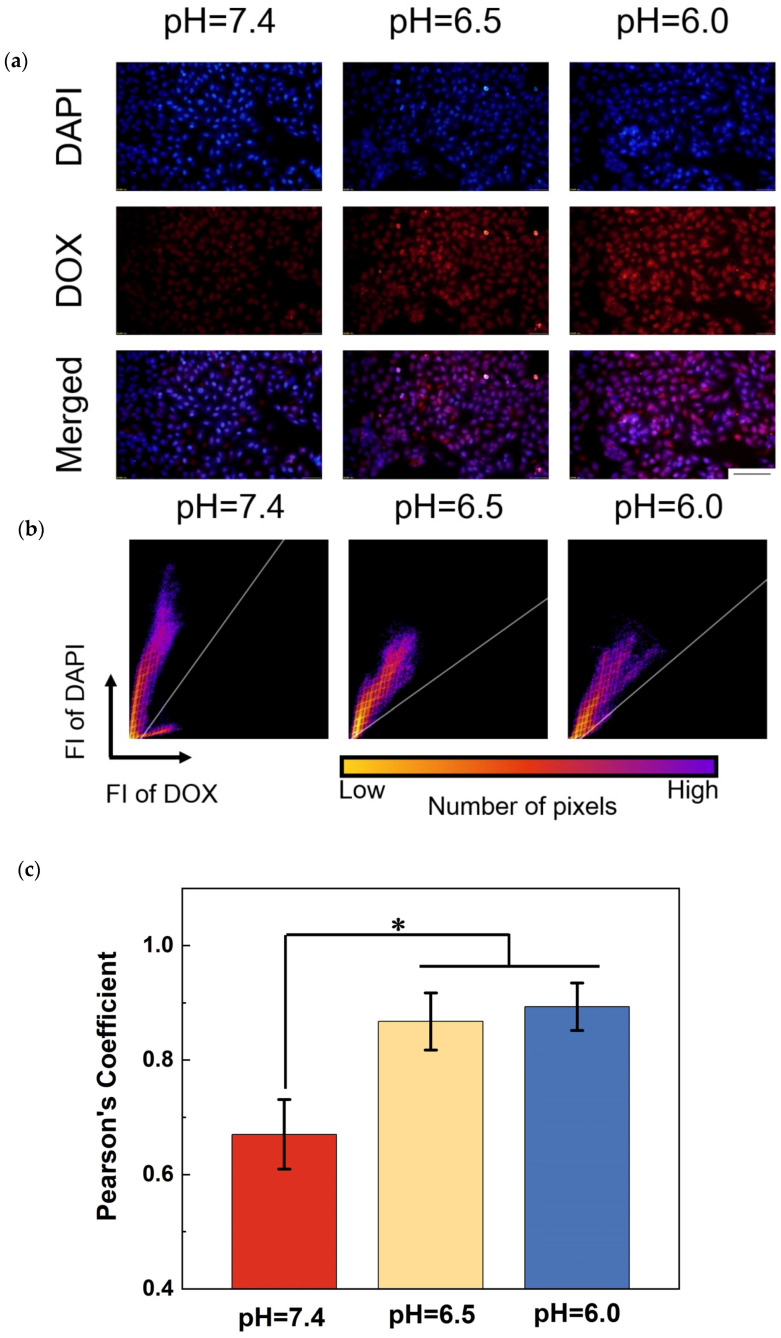
Evidence for the pH-sensitive co-localization of MG63 nuclei and DOX released from TNA-PDA-DOX after indirect culture for 6 h. (**a**) Overlays of confocal microscopic images of MG63 cells stained by DAPI and DOX, scale bar: 50 μm; (**b**) Representative two-dimensional fluorescence intensity histograms of stained nuclei and DOX; (**c**) Fluorescence intensity co-localization (Pearson’s coefficient) at pH = 7.4, 6.5, and 6.0. *: *p* < 0.05.

To visualize the intracellular localization of DOX released from TNA-PDA-DOX samples at different pH values after indirect culture with MG63 cells for 6 h, a co-localization analysis using DAPI for nuclei staining was performed as presented in Figure 10. Figure 10a shows the confocal fluorescence micrographs of the MG63 nuclei (blue) and DOX (red) released from the TNA-PDA-DOX group at pH = 7.4, 6.5, and 6.0. The slightly acidic environment leads to a strong accumulation of DOX in the nucleus, which is clearly indicated by the purple spots in the merged images, resulting from the co-localization of blue and red signals. Furthermore, the confocal images were quantitatively analyzed to generate two-dimensional fluorescence intensity histograms, and the Pearson correlation coefficients (i.e., *R*) were also calculated accordingly. Pearson’s coefficient *R*, ranging from −1 to 1, describes the correlation of the intensity distribution between the two channels. The values 1, 0, and −1 represent a total positive correlation, no correlation, and a total negative correlation, respectively. A channel of DAPI was used versus the DOX channel to obtain histograms, and representative results at the three pH values are shown in Figure 10b. Single population groups are observed on the histograms of the pH = 6.5 and 6.0 configurations. In contrast, pixels on the histograms of the pH = 7.4 group are separated into two groups, indicating relatively poor co-localization. Figure 10c shows that the calculated *R* values are about 0.8–0.95 for the two acidic configurations, whereas that at a neutral pH is only around 0.7.

## 4. Discussion

LDD is now considered a promising way to avoid major limitations of systemic chemotherapy, such as widespread toxicity and insufficient drug concentrations reaching the tumor [19,44]. In the present work, a pH-responsive Ti-based LDD prototype was proposed, and DOX was selected as a prototypical model of anti-cancer agents. Since PDA usually shows good biocompatibility, great adhesivity onto almost any solid surface, and protonation-dependent solubility [45], it was introduced as an intermediate layer between TNAs and DOX for both efficient immobilization and pH-responsive release of DOX molecules.

The FTIR spectra (see Figure 2) show that PDA and DOX were successfully loaded onto the TNAs. The SEM images presented in Figure 3 show that the introduction of PDA significantly reduced the diameter of nanotubes, indicating that the PDA coating tends to adhere to the inner walls of nanotubes rather than simply cover the surface of TNAs. Such a result is consistent with the result reported by Yang et al. [24]. Furthermore, De Santo found that over 90% of DOX dwelled preferentially in inter-nanotube spaces, so the DOX loading did not change the surface morphology in this work.

Considering that the first contact of implants with surrounding tissues starts from the displacement of body fluid from the interface, the wettability can determine the cell response [16]. Furthermore, it is believed that a more hydrophobic surface of TNAs is beneficial in prolonging the release of hydrophilic drugs [46]. Therefore, it is important to analyze the influence of surface treatment on the hydrophilicity of implants. As shown in Figure 4, the introduction of PDA on TNAs leads to a significant decrease in hydrophilicity, because PDA is less hydrophilic than Ti(OH)_4_ formed on the surface of TNAs [16,47]. After the loading of DOX, the final contact angles of TNA-PDA-DOX samples are almost the same as those of TNA-DOX, which should probably be related to the good aqueous solubility of DOX [48]. Consequently, it is reasonable to assume that the coatings were successfully assembled in the sequence of PDA covered with DOX on the inside walls of TNAs.

DOX is listed as one of the most effective chemotherapeutic medications, but its high distribution throughout the body can be harmful to healthy cells by causing myelosuppression, cardiotoxicity, and nephrotoxicity [49]. Therefore, suitable DOX drug delivery systems are needed. The low pH is usually recognized as a universal diagnostic hallmark of cancer, so a pH-responsive delivery system of chemotherapeutic agents is of particular interest for cancer theranostics [50]. It has been reported that the solubility of DOX increases in solutions with lower pH values. However, there is no significant difference among the drug release behavior from TNA-DOX at any pH conditions as shown in Figure 5a,c, indicating that DOX is not so sensitive for a pH-responsive TNA-based LDD system. On the contrary, after employing PDA as an intermediate layer, the TNA-PDA-DOX samples exhibit significant pH-responsive drug release behavior as presented in Figure 5b,d.

It is important to point out that although it has been reported that low pH values may result in the degradation of PDA on nanoparticles [51], the extremely low amount of DA release from the PDA-coated samples (see Figure S1) verifies the stability of the PDA coating in the slightly acidic environment in this work.

Based on the simulation data presented in Figure 6 and Table 1, the lower *k_1_* and *k_2_* of TNA-PDA-DOX than those of TNA-DOX at the same pH value indicate a more controllable drug release in the former. Furthermore, the significant differences between the *k_1_* values at different pH values also suggest the obvious pH-responsive release behavior of TNA-PDA-DOX. Compared to previous Ti-based LDD systems, this TNA-PDA-DOX system achieves stability under physiological pH conditions through π-π stacking interactions between DOX molecules and PDA coating [52]. In typical slightly acidic tumor environments, the increased protonation disrupts these interactions, leading to drug release. Therefore, this design enables effective pH-sensitive DOX release.

Biosafety is always essential for metallic biomaterials [53]. In this work, the cytotoxicity of MC3T3 cells was tested by direct culture. The significant difference in DOX cumulative release from TNA-PDA-DOX and TNA-DOX at neutral environment at 24 h (see Figure 5) should account for the acceptable cell viability of the former and the poor biocompatibility of the latter (see Figure 7a). The cell morphology images presented in Figure 7b exhibit preferred cell spreading on TNA-PDA-DOX, TNA-PDA, and CP-Ti rather than on TNA-DOX. Furthermore, the significantly higher numbers of adhered live MC3T3 cells (see Figure 7c) and an order of magnitude lower percentage of dead cells (see Figure 7d) on TNA-PDA-DOX compared with those on TNA-DOX should be ascribed to the significantly different DOX release behavior from the two configurations at neutral environment (see Figure 5). These results verify the great biocompatibility enhancement after introducing the intermediate PDA layer between TNAs and DOX.

The anti-cancer behavior is the most important biofunction in this work, so both direct and indirect culture modes were employed for the viability assessments of MG63 cells. The direct culture simulates the in vivo situation where the tumor cells directly contact the implant. Considering the similar nanotubular surface morphology (see Figure 3) as well as the good hydrophilicity (see Figure 4) of TNA-PDA, TNA-PDA-DOX, and TNA-DOX, the significantly different cell responses (see Figure 8) on these configurations should be mainly attributed to the DOX released from the latter two samples. It is interesting that the MG63 cells cultured on TNA-PDA-DOX and TNA-DOX exhibit comparable viability as presented in Figure 8, which is quite different from the cell response of MC3T3 on the two configurations as shown in Figure 7. In the direct culture experiments, cells were directly seeded onto the samples, and thus the microenvironment around the cells can significantly influence the drug release from the samples. As reported by Hu et al. [54], carbonic anhydrase IX (CA-IX) catalyzes the carbon dioxide (CO_2_), produced by the mitochondrial respiration in tumor cells, into bicarbonate ions (HCO_3_^−^) and protons (H^+^), and then extrudes H^+^ into the extracellular environment, leading to acidification of the microenvironment. The expression of CA-IX in MG63 cells can be induced under normoxic conditions within the in vitro culture environment [55]. Therefore, the microenvironment around MG63 cells is acidic, resulting in the enhanced release of DOX from TNA-PDA-DOX (see Figure 8). However, CA-IX is not expressed in MC3T3 cells [56], so the good cell viability of MC3T3 on TNA-PDA-DOX (see Figure 7) should be attributed to the neutral microenvironment of MC3T3 cells.

The indirect culture is designed to show the influence of drug release from the implant on surrounding tumor cells that do not directly contact the implant, so the cell response is mainly due to the contents of the culture medium. It is important to note that the cell viabilities of MG63 on both CP-Ti and TNA-PDA at pH = 7.4, 6.5, and 6.0 (see Figure 9) do not show significant differences, indicating that the cell response of MG63 can hardly be directly influenced by the medium environment with pH ranging from 6.0 to 7.4. However, the cell viability of the TNA-PDA-DOX group is highly pH-dependent. Such a result should be mainly owing to the pH-responsive release of DOX. Furthermore, the DOX co-localization results as shown in Figure 10 indicate higher accumulations of DOX in the nuclei of MG63 at pH = 6.5 and 6.0 than that at pH = 7.4, suggesting that the slightly acidic environment might account for the improved uptake of DOX into cancer cells.

## 5. Conclusions

In this work, a pH-responsive anti-cancer local drug delivery prototype (i.e., TNA-PDA-DOX) was developed. The results suggest that TNA-PDA-DOX shows a regular nanotubular-structured surface, good hydrophilicity, and pH-dependent DOX release behavior. The low amount of DOX release from TNA-PDA-DOX at neutral environment ensures its good biocompatibility. However, TNA-PDA-DOX exhibits obvious anti-cancer behavior when directly or indirectly cultured with osteosarcoma cell line MG63, because the acidic extracellular environment can significantly enhance DOX release. Although this pH-responsive implant demonstrates its great potential as a prototype for the clinical treatment of osteosarcoma, more work should be performed to achieve better controllable and sustained drug release in the future.

## Figures and Tables

**Table 1:** Kinetic parameters for the DOX release from TNA-DOX and TNA-PDA-DOX at pH = 7.4, 6.5, or 6.0 (Obtained from fitting the experimental data to Equation (5)).

	**TNA-DOX**		**TNA-PDA-DOX**	
	*k_1_*	*k_2_*	*k_1_*	*k_2_*
pH = 7.4	0.199	0.012	0.070	0.008
pH = 6.5	0.283	0.020	0.182	0.012
pH = 6.0	0.307	0.022	0.195	0.016

## Data Availability

The data that support the findings of this work are available on request from the corresponding author. The data are not publicly available due to privacy or ethical restrictions.

## Supplementary Materials

The following supporting information can be downloaded at: https://www.mdpi.com/article/10.3390/jfb15100312/s1, Figure S1: The cumulative DA release from (a) TNA-PDA and (b) TNA-PDA-DOX at pH = 6.0 for 24, 48, and 72 h.
