# Supplementary figures and images for: A pH-Responsive Ti-Based Local Drug Delivery System for Osteosarcoma Therapy
Source: J Funct Biomater. 2024 Oct 21;15(10):312. doi: 10.3390/jfb15100312 (PMC11508615; doi:10.3390/jfb15100312)

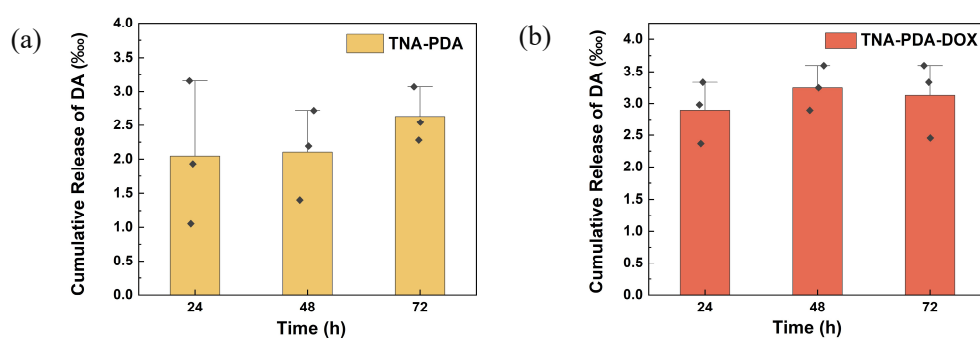

**Figure S1.** The cumulative DA release from (a) TNA-PDA and (b) TNA-PDA-DOX at pH=6.0 for 24, 48, and 72 h.

Supplement: Supplementary file 1 [file jfb-15-00312-s001.zip › jfb-3191601-supplementary.pdf]
